# RETRACTION: The Role of Traditional Chinese Medicine in Postoperative Wound Complications of Gastric Cancer

**DOI:** 10.1111/iwj.70660

**Published:** 2025-04-23

## Abstract

**RETRACTION:** ChenS. , TianX. , LiS. , WuZ. , LiY. , GuoJ. , and LiaoZ. , “The Role of Traditional Chinese Medicine in Postoperative Wound Complications of Gastric Cancer,” International Wound Journal 21, no. 4 (2024): e14847, 10.1111/iwj.14847.38584331 PMC10999554

The above article, published online on 07 April 2024, in Wiley Online Library (http://onlinelibrary.wiley.com/), has been retracted by agreement between the journal Editor in Chief, Professor Keith Harding; and John Wiley & Sons Ltd. Following an investigation by the publisher, all parties have concluded that this article was accepted solely on the basis of a compromised peer review process. The editors have therefore decided to retract the article.

**RETRACTION:** S. Chen , X. Tian , S. Li , Z. Wu , Y. Li , J. Guo , and Z. Liao , “The Role of Traditional Chinese Medicine in Postoperative Wound Complications of Gastric Cancer,” International Wound Journal 21, no. 4 (2024): e14847, 10.1111/iwj.14847.38584331 PMC10999554

The above article, published online on 07 April 2024, in Wiley Online Library (http://onlinelibrary.wiley.com/), has been retracted by agreement between the journal Editor in Chief, Professor Keith Harding; and John Wiley & Sons Ltd. Following an investigation by the publisher, all parties have concluded that this article was accepted solely on the basis of a compromised peer review process. The editors have therefore decided to retract the article.

